# Structural basis for Ccd1 auto-inhibition in the Wnt pathway through homomerization of the DIX domain

**DOI:** 10.1038/s41598-017-08019-5

**Published:** 2017-08-10

**Authors:** Shin-ichi Terawaki, Shohei Fujita, Takuya Katsutani, Kensuke Shiomi, Kazuko Keino-Masu, Masayuki Masu, Kaori Wakamatsu, Naoki Shibata, Yoshiki Higuchi

**Affiliations:** 1 Graduate School of Science and Technology, Gunma University, 1-5-1 Tenjin-cho, Kiryu, Gunma, 376-8515 Japan; 2 RIKEN SPring-8 Center, 1-1-1 Koto, Mikazuki-cho, Sayo-gun, Hyogo, 679-5248 Japan; 3 Department of Life Science and Department of Picobiology, Graduate School of Life Science, University of Hyogo, 3-2-1 Koto, Kamigori-cho, Ako-gun, Hyogo, 678-1297 Japan; 4 Department of Molecular Neurobiology, Faculty of Medicine, University of Tsukuba, 1-1-1 Tennodai, Tsukuba, Ibaraki, 305-8575 Japan

## Abstract

Wnt signaling plays an important role in governing cell fate decisions. Coiled-coil-DIX1 (Ccd1), Dishevelled (Dvl), and Axin are signaling proteins that regulate the canonical pathway by controlling the stability of a key signal transducer β-catenin. These proteins contain the DIX domain with a ubiquitin-like fold, which mediates their interaction in the β-catenin destruction complex through dynamic head-to-tail polymerization. Despite high sequence similarities, mammalian Ccd1 shows weaker stimulation of β-catenin transcriptional activity compared with zebrafish (z) Ccd1 in cultured cells. Here, we show that the mouse (m) Ccd1 DIX domain displays weaker ability for homopolymerization than that of zCcd1. Furthermore, X-ray crystallographic analysis of mCcd1 and zCcd1 DIX domains revealed that mCcd1 was assembled into a double-helical filament by the insertion of the β1-β2 loop into the head-to-tail interface, whereas zCcd1 formed a typical single-helical polymer similar to Dvl1 and Axin. The mutation in the contact interface of mCcd1 double-helical polymer changed the hydrodynamic properties of mCcd1 so that it acquired the ability to induce Wnt-specific transcriptional activity similar to zCcd1. These findings suggest a novel regulatory mechanism by which mCcd1 modulates Wnt signaling through auto-inhibition of dynamic head-to-tail homopolymerization.

## Introduction

The Wnt signaling pathway plays key roles in cell fate determination during embryonic development, neurogenesis, homeostasis, and oncogenesis^1–4^. In the canonical pathway, the Wnt-receptor complex controls the stability of a key effector β-catenin by regulating the activity of a cytoplasmic β-catenin destruction complex^5^. In the absence of Wnt signaling, the β-catenin destruction complex mediates β-catenin phosphorylation by serine/threonine protein kinases, glycogen synthase kinase 3β (GSK3β) and casein kinase 1α (CKα), which are regulated by two scaffold proteins, Axin and adenomatous polyposis coli (APC)^6–10^. The phosphorylated β-catenin is then degraded by the ubiquitin-proteasome system^11, 12^. In the presence of Wnt signaling, signal transducers Dishevelled (Dvl) and Coiled-coil-DIX1 (Ccd1) are activated through Wnt ligand-receptor interaction and suppress β-catenin destruction by binding to Axin, thus promoting the translocation of unphosphorylated β-catenin to the nucleus and stimulating the transcription of Wnt target genes^13–17^.

Three proteins, Dvl, Ccd1, and Axin, play an important role in β-catenin-mediated transcription through formation of a transient molecular complex which is translocated to the plasma membrane in the canonical Wnt pathway^15, 18–22^. In response to stimulation by Wnt signaling, these proteins can form dynamic polymers^19, 23^ observed as cytoplasmic puncta in various species *in vivo* ^20, 24–29^ as well as in cell cultures upon overexpression^18, 20, 21, 30, 31^. The formation of cytoplasmic puncta has been shown to correlate with the ability to activate the canonical Wnt signaling pathway^32, 33^, possibly through interaction with and activation of transmembrane receptors Frizzled and LRP6, as shown *in vitro* for Dvl and Axin^23^. Thus, dynamic polymerization of Dvl, Ccd1, and Axin proteins provides an interaction platform with other factors of the Wnt pathway, resulting in the activation of the downstream signaling^34^.

The polymerization of Dvl, Ccd1 and Axin, which depends on the intracellular concentration, is mediated by their terminal DIX domains^17, 32, 33^. These domains form helical filaments engaged in homomeric head-to-tail interactions via a parallel intermolecular β-bridge between β2 and β4 strands in the ubiquitin-like fold structure comprising five β-strands and one α-helix^17, 32^. Mutations in the DIX domain disrupt the homomeric head-to-tail interaction and formation of cytoplasmic puncta, and inhibit β-catenin-dependent transcription^32, 33^. Furthermore, Dvl, Ccd1, and Axin can directly interact with each other through their DIX domains which form the same head-to-tail structures^17, 33^. These findings suggest that the DIX domain possesses versatility as a double-faced signaling module which can provide both homomeric and heteromeric interactions^35^.

Although the functional importance of dynamic head-to-tail polymerization via DIX domain has been previously studied, the precise mechanism of DIX-mediated homomeric polymerization and its functional significance in regulating Wnt downstream signaling is unknown. Previously, we reported that mouse Ccd1 (mCcd1) showed low level of transcriptional activation compared to zebrafish Ccd1 (zCcd1), suggesting that mCcd1 signaling activity is controlled by homopolymerization mode distinct from those of other DIX-containing proteins^36^. Here, we report crystal structures of DIX domains in mCcd1 and zCcd1 resolved without additional mutagenesis or heavy atom modification. Interestingly, we found that the DIX domain of mCcd1 presented double-helical filaments formed by two head-to-tail helical polymers in crystal, whereas DIX domains of zCcd1 had a head-to-tail helical filament structure similar to that of the Hg-modified Axin DIX domain reported previously^32^. Point-directed mutagenesis of predicted structurally important residues followed by binding assays and transcriptional activity measurements revealed that loop β1-β2 of the mCcd1 DIX domain inserted into head-to-tail interface prevented the formation of head-to-tail helical filaments and, consequently, inhibited mCcd1 transcriptional activity. These results disclose a novel regulatory mechanism of Wnt signaling in mammals through conformational auto-suppression of mCcd1, suggesting that the DIX domain functions as a switching hub in the canonical Wnt pathway via dynamic polymerization.

## Results

### mCcd1 DIX domain has a low affinity to dynamic homopolymerization

**Figure 1:**
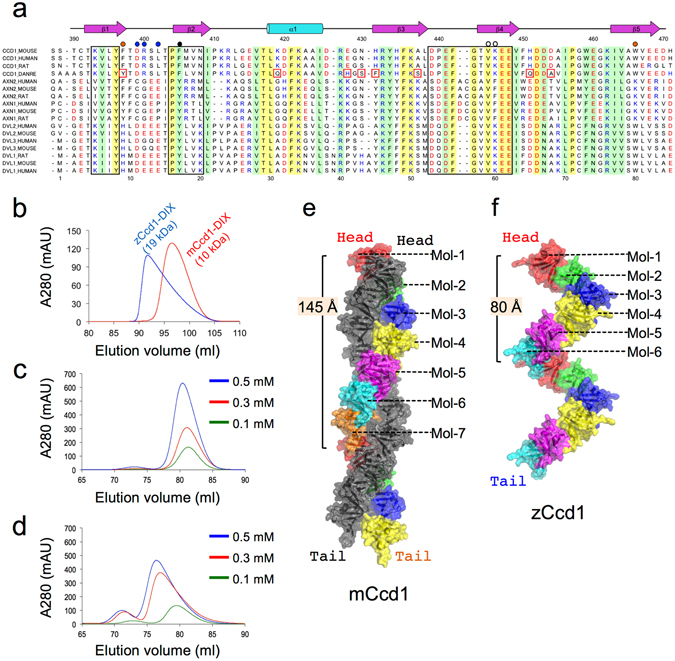
Hydrodynamic properties and structures of helical homopolymers formed by mCcd1 and zCcd1 DIX domains. (**a**) Amino acid sequence alignment of DIX domains from Ccd1, Axin, and Dvl; secondary structural elements of mCcd1 are shown at the top. Conserved and semi-invariant residues (E = D, R = K = H, T = S, F = Y, V = L = I = M = C) are highlighted yellow and green, respectively, and acidic and basic residues are marked red and blue, respectively. Amino acids predicted to be involved in head-to-tail interactions are outlined by black boxes. The black and white circles indicate positions of M4 and M2 mutations, respectively. Residues unique in zCcd1 are shown in red boxes, and those involved in double-helical polymerization are marked by blue and brown circles. (**b**–**d**) Hydrodynamic properties of mCcd1 and zCcd1 DIX domains. Gel filtration profiles of DIX domains of zCcd1 and mCcd1 (**b**). Gel filtration profiles of the MBP-fused mCcd1 DIX (**c**) and the MBP-fused zCcd1 DIX (**d**) at different loading concentrations. (**e**,**f**) Structures of mCcd1 double-helical (**e**) and zCcd1 single-helical (**f**) homopolymers. Each molecule forming one helical turn in helical filaments is shown in different color. Another helical filament in the mCcd1 double-helical homopolymer is shown in black. Pitch lengths of mCcd1 and zCcd1 helical polymers were ~145 and ~80 Å, respectively.

Multiple amino acid sequence alignment of DIX domains of Ccd1, Dvl, and Axin showed that they had about 30% sequence identity (Fig. 1a). The key residues involved in the homomeric head-to-tail polymerization of the DIX domain are highly conserved in these proteins. However, the zCcd1 DIX domain contains unique amino acids different from characteristic residues conserved in DIX domains of human and mouse Ccd1 (showed by red boxes in Fig. 1a). These findings suggest a possibility that differences in DIX domain sequences confer distinct activation properties.

Hydrodynamic properties of mCcd1 and zCcd1 DIX domains were characterized by gel filtration chromatography. The mCcd1 DIX domain was mainly eluted as a monomer with a molecular mass of 10 kDa, whereas the zCcd1 DIX domain was eluted as a dimer with an apparent molecular mass of 19.0 kDa with its monomer included as a minor state in the elution peak (Fig. 1b). In addition, we examined whether the DIX domains aggregated into larger homomeric complexes in a concentration-dependent manner. Since the zCcd1 DIX domain has low solubility, maltose-binding protein (MBP)-fused DIX domains were used to prepare high-concentration solutions. In these conditions the MBP-fused mCcd1 DIX did not form high-molecular-weight polymers (Fig. 1c), suggesting a similar molecular behavior to the human Ccd1 (hCcd1) DIX domain^17^. In contrast, the MBP-fused zCcd1 DIX demonstrated a shift to higher molecular weight in a concentration-dependent manner (Fig. 1d), indicating similarity with other DIX-containing proteins, Dvl and Axin^32, 33^. These results suggest that, despite high sequence similarity with the zCcd1 DIX domain, the mCcd1 DIX domain has unique hydrodynamic properties defining its weak ability to dynamic homopolymerization.

### Structure determination of mCcd1 and zCcd1 DIX domains

To obtain crystals of mCcd1 and zCcd1 DIX domains, they were expressed as recombinant proteins (residues 388–470 and 356–438, respectively; Fig. 1a). First, we obtained mCcd1 DIX crystals in solution containing polyethylene glycol (PEG) 3350 as precipitant, although the crystals diffracted poorly. The crystals suitable for structural analysis were obtained by additional screening using commercial screening kits containing glycerol as an additive^37^. Structures of mCcd1 and zCcd1 DIX domains were determined by multiple wavelength anomalous dispersion (MAD) using selenomethionine-substituted protein crystals and a molecular replacement method with the mCcd1 DIX domain as a search model, respectively. Subsequently, mCcd1 and zCcd1 structures were refined at 3.00 Å resolution to the *R*_work_ value of 24.8% (*R*_free_, 28.9%) and at 1.96 Å resolution to the *R*_work_ value of 16.9% (*R*_free_, 21.6%), respectively (Table 1). The crystallized DIX domains contained seven (mCcd1) and one (zCcd1) molecules per asymmetric unit. The mCcd1 DIX domains in the asymmetric unit had essentially the same structure with an average root-mean-square (RMS) deviation of 0.55 Å for 391–468 residues.

**Table 1:** Data collection and refinement statistics.

	Mouse CCD1 Native	Mouse CCD1 selenomethionine derivative			Zebrafish CCD1
		Peak	Edge	Remote	
**Data collection**					
Space group	*P*2_1_2_1_2_1_	*P*2_1_2_1_2_1_	*P*2_1_2_1_2_1_	*P*2_1_2_1_2_1_	*P*6_1_
Cell dimensions					
*a*, *b*, *c* (Å)	72.9, 75.7, 125.6	72.9, 78.6, 125.9	72.9, 78.6, 125.9	72.9, 78.7, 125.9	46.7, 46.7, 79.4
α, β, γ (°)	90, 90, 90	90, 90, 90	90, 90, 90	90, 90, 90	90, 90, 120
Wavelength (Å)	1.0000	0.9748	0.9793	0.9641	1.1000
Resolution (Å)	50.0–3.00	50.0–3.20	50.0–3.20	50.0–3.20	50.0–1.96
	(3.11–3.00)	(3.31–3.20)	(3.31–3.20)	(3.31–3.20)	(1.98–1.96)
Reflection					
Measured	89,162	148,041	148,755	148,091	63,615
Unique	14,312	11,893	11,857	11,848	7,076
Redundancy^a^	6.2 (4.5)	12.5 (8.4)	12.6 (8.6)	12.5 (8.7)	9.0 (8.4)
Completeness^a^ (%)	99.0 (91.6)	95.5 (72.5)	95.2 (70.1)	95.0 (68.6)	99.1 (97.9)
Mean *I*/σ(*I*)^a^	16.3 (2.3)	15.3 (2.0)	17.0 (2.2)	14.5 (2.2)	35.3 (5.0)
*R*_merge_^a^ (%)	10.7 (52.8)	14.7 (54.7)	13.8 (51.2)	14.4 (54.8)	12.7 (65.4)
*R*_mean_ (%)					13.5 (69.6)
*R*_pim_ (%)					4.5 (23.6)
**Refinement**					
Resolution (Å)	29.1–3.00				23.4–1.96
*R*_work_/*R*_free_^b^ (%)	24.8/28.9				16.9/21.6
No. of atoms					
Protein	4665				734
Water	—				66
*Mean B* factors (*Å* ^2^)					
Protein	58.4				38.2
Water	—				43.3
r. m. s. deviations					
Bond lengths (Å)	0.003				0.004
Bond angles (°)	0.798				0.757

^a^Numbers in parentheses refer to statistics for the outer resolution shell.

^b^ *R*_work_ = Σ| |*F*_obs_| − |*F*_calc_| |/|*F*_obs_|. *R*_free_ is the same as *R*_work_ except that a 5% subset of all reflections was held aside throughout the refinement.

### Overall structures of helical polymers formed by DIX domains

DIX domains of mCcd1 and zCcd1 are arranged into head-to-tail helical filaments by parallel β-bridge interactions (Fig. 1e, f). Interestingly, the mCcd1 DIX domain forms a unique double-helical filament with seven molecules per turn along the crystallographic 2_1_-axis with a pitch of ~145 Å, in spite of weak ability for homopolymerization (Fig. 1e). Two left-hand helical filaments engaged in homomeric head-to-tail interactions are arranged parallel to each other. In contrast, the zCcd DIX domain is assembled into single-helical filament-like structures consisting of six or seven molecules per turn with a pitch of ~80 Å (Fig. 1f). The N-terminal residues of the DIX domains are directed toward outside in both single- and double-helical filaments, suggesting that the N-terminal domains of Ccd1 proteins do not interfere directly with the DIX-homopolymerization.

The crystal structure of the hCcd1 DIX domain (98% sequence identity with the mCcd1 DIX domain) has been reported by Liu *et al*.^17^. The hCcd1 DIX domain formed single-helical filaments containing six molecules per turn, indicating structural similarity to zCcd1 DIX, although hCcd1 DIX has hydrodynamic properties in solution similar to mCcd1 DIX but not to zCcd1 DIX (Fig. S1a,b). The structural difference between hCcd1 and mCcd1 DIX homopolymers could be caused by interactions with crystallographically symmetric molecules through four artificial residues at the N-terminus remaining from the expression vector, which are absent in the mCcd1 DIX construct (Fig. S1c,d). These extra residues added to hCcd1 DIX could have an effect on the head-to-tail interaction between DIX domains through contact with strand β4 and loop β4-β5 from the two-fold axis symmetric head-to-tail dimer in crystals (Fig. S1e). However, as Liu *et al*. reported previously, these extra residues are unlikely to interfere with the DIX-homopolymerization in solution, because of the low binding affinity to the contact site observed in crystal^17^. The structural comparison of mCcd1 and zCcd1 DIX-homopolymers with hCcd1 indicates the disagreements between the behavior of the hCcd1 DIX domain in solution and the homopolymer structure. Therefore, structural analysis of the mCcd1 homopolymer without artificial sequence could provide valuable clues for understanding decreased ability for dynamic homopolymerization in mammalian Ccd1 DIX.

### Structures of DIX domains and head-to-tail interactions

The mCcd1 and zCcd1 DIX domains have a ubiquitin-like fold with five β-strands and one short α-helix (Fig. S2a,b). The average overall RMS deviation between DIX domains is 1.43 Å for 80 Cα-carbon atoms (Fig. S2c–e). Structural comparison revealed conformational changes in the four connecting loops of the secondary structures, except for loop β4-β5. Significant deviation is found in the β3-β4 region which in the DIX domains is formed by a type I β-turn comprising five residues (439–443 and 407–411, respectively), whereas the Axin DIX domain has a long loop of seven residues (798–804) (Fig. 1a). Similarly, the DIX domains in mCcd1 and the Dvl1 Y17D mutant also show structural resemblance with an overall RMS deviation of 1.04 Å (Fig. S2e). Significant conformational changes in the α1-β3 loops are obviously caused by two inserted residues.

**Figure 2:**
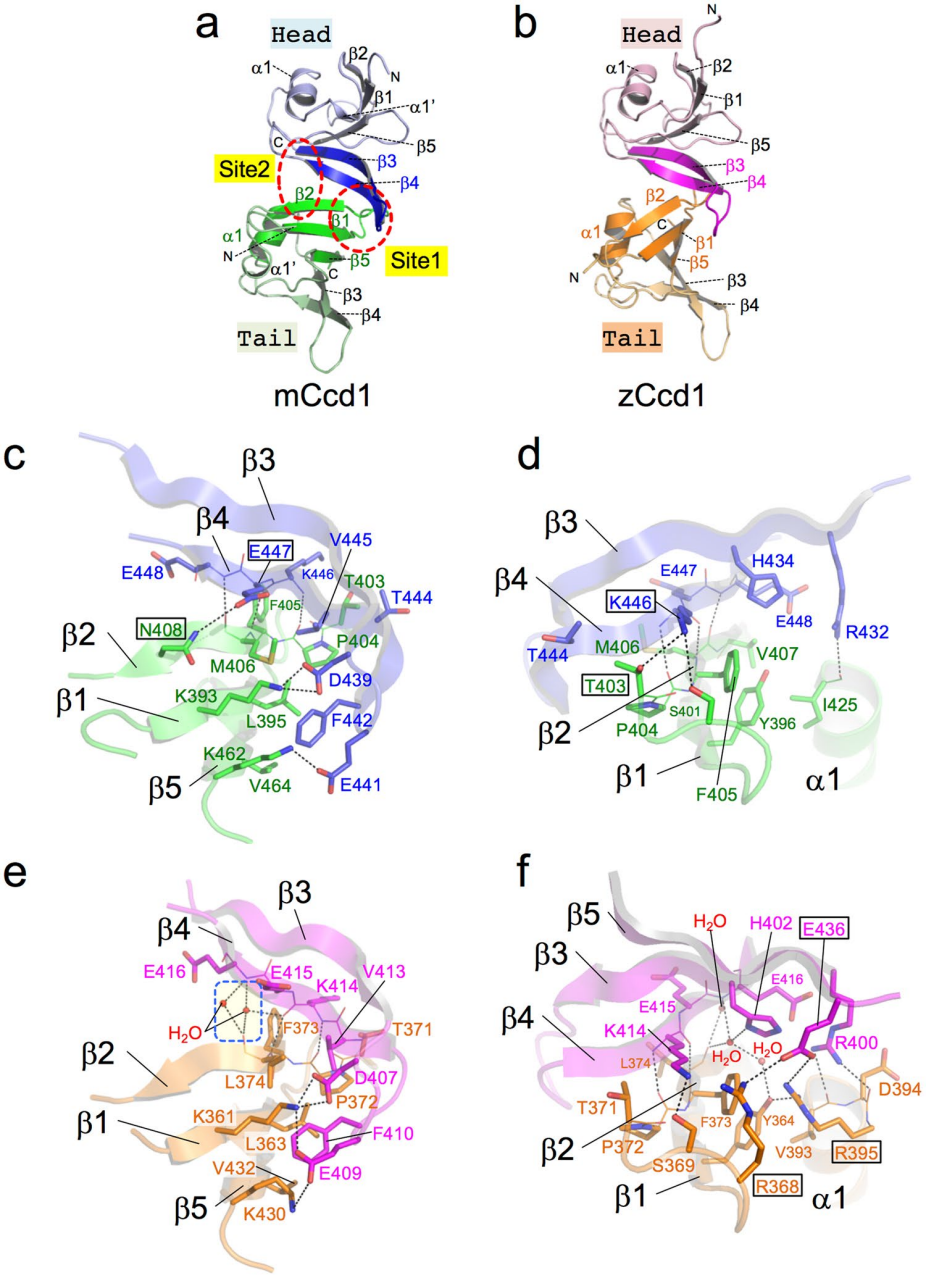
Head-to-tail interaction in Ccd1 helical homopolymers. (**a**,**b**) Structures of head-to-tail helical homodimers. The head and tail regions in homodimeric interfaces are colored blue and green in mCcd1 (**a**), magenta and orange in zCcd1 (**b**), respectively. Contact sites 1 and 2 in the head-to-tail interface of mCcd1 are indicated by red dashed circles. (**c**,**d**) Close up views of Site 1 (**c**) and Site 2 (**d**) in mCcd1 homodimer. The head and tail regions are colored blue and green in mCcd1. Hydrogen bonds and salt bridges are indicated by black dashed lines. Residues whose side chains participate in unique hydrogen bond are labeled with boxes. (**e**,**f**) Close up view of Site 1 (**e**) and Site 2 (**f**) in zCcd1. The head and tail regions are colored magenta and orange. Water-mediated hydrogen bonds between main chains are boxed by a blue dashed line. Residues whose side chains participate in unique electrostatic interactions are labeled with boxes.

Homomeric head-to-tail interactions between Ccd1 DIX domains occurred in the molecular interface created by β-sheet β3-β4 of one monomer and β-sheet β2-β1-β5 of the other (Fig. 2a, b). The β4 strand of one monomer forms a parallel β-bridge with the β2 strand of the adjacent monomer through three hydrogen bonds between main chains. The average total accessible surface area of homomeric head-to-tail interface is 592.2 Å^2^. Strands β3 and β4 in the mCcd1 DIX domain creates two contact sites (Site 1 and Site 2) with the adjacent monomer (Fig. 2a). In Site 1, the side chains of two acidic residues, Asp439 and Glu441, are located within a distance of a salt bridge from the side chains of Lys393 (strand β1) and Lys462 (strand β5), respectively (Fig. 2c). Glu447 (strand β4) forms a hydrogen bond with Asn408 (strand β2). In addition, the side chains of Phe442 (strand β3) and Val445 (strand β4) contact the hydrophobic surface created by three residues, Leu395, Met406, and Val464, from the β2-β1-β5 sheet. In contact Site 2, the side chain of Lys446 forms hydrogen bonds with Ser401 and Thr403 (loop β1-β2) of the adjacent monomer (Fig. 2d), while helix α1 is capped at the C-terminus by the side chain of Arg432 (strand β3) through a hydrogen bond with the main chain carbonyl group of Ile425.

In the zCcd1 dimer, homomeric interaction is similar to that of mCcd1 described above (Fig. 2e and f). Consistent with this, the accessible surface area in the homomeric head-to-tail interface of the zCcd1 dimer is 590.7 Å^2^. In addition, the interface of the zCcd1 dimer has two water-mediated hydrogen bonds (Fig. 2e). In Site 2 of zCcd1, Glu436 (strand β5) in the head-binding molecule have two characteristic electrostatic interactions with Arg368 and Arg395 (Fig. 2f); however, no interaction is observed at the same position in the mCcd1 dimer. Moreover, the side chain of His402 (strand β3) in the head-binding molecule forms hydrogen bonds with Tyr364 via two water molecules. These results suggest that unique interactions observed in zCcd1 may contribute to stabilization of the single-helical polymer.

### The formation of double-helical structure may be responsible for the low ability of mCcd1 DIX to homopolymerize

**Figure 3:**
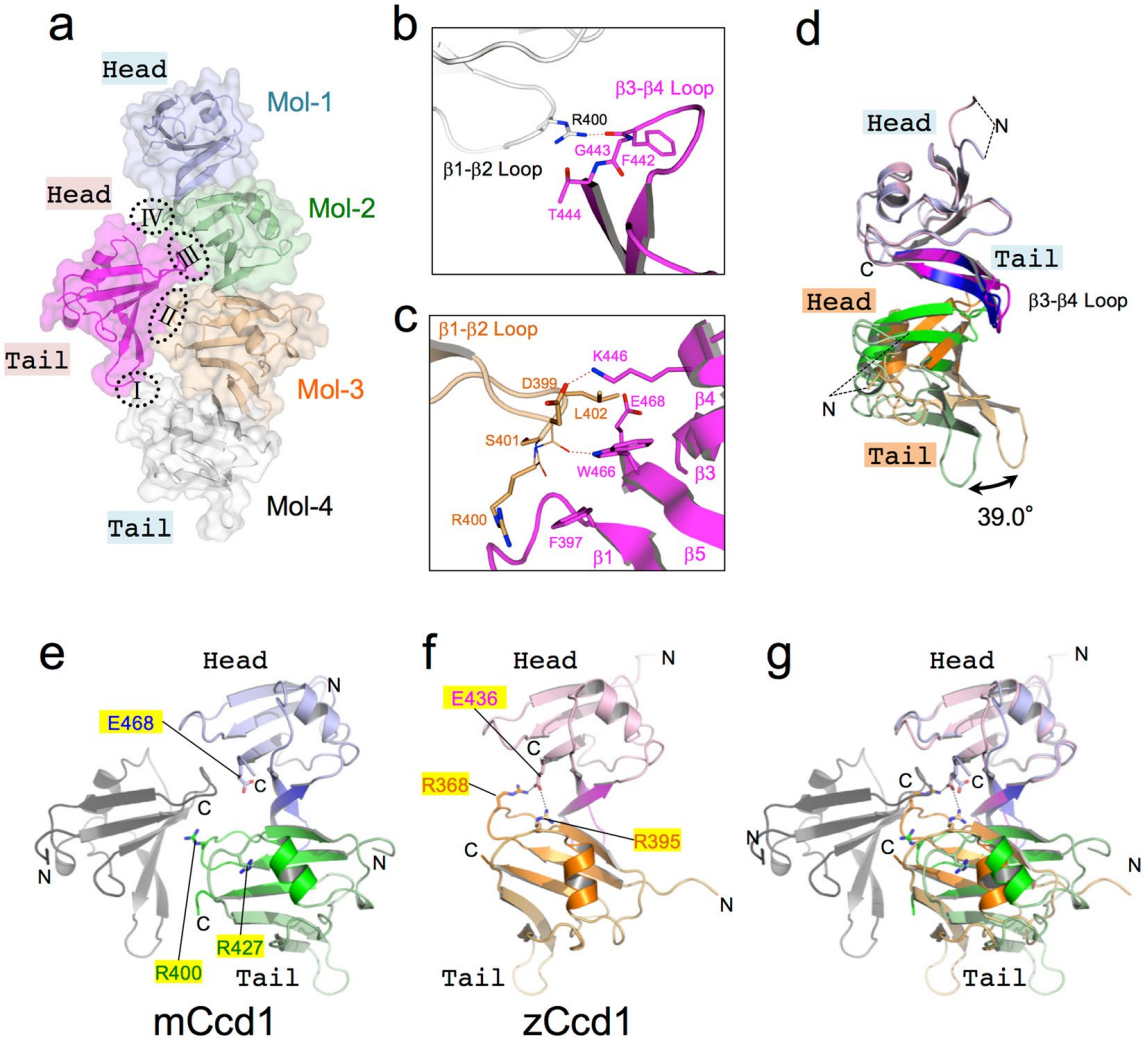
Molecular interactions in the mCcd1 double-helical homopolymer. (**a**) Contact interfaces. The mCcd1 DIX domain (magenta) has contact interfaces with four molecules (blue, green, orange, and white) of another intertwined head-to-tail helical filament. Contact sites I and IV are created between loops β1-β2 and β3-β4, and contact sites II and III are formed by the interaction of loop β1-β2 with β-sheet β1-β5-β3-β4. (**b**,**c**) Close up view of contact site I (**b**) and II (**c**). Residues involved in the formation of the contact site are shown as stick models. Electrostatic bonds are indicated by dashed lines. (**d**) Superposition of mCcd1 and zCcd1 homodimers. Head-to-tail interfaces are colored blue and green in mCcd1, and magenta and orange in zCcd1, respectively. The resultant angular differences are indicated by a double-headed arrow. (**e**) Interference of the mCcd1 double-helical homopolymer with electrostatic bond formation in the head-to-tail interface. The mCcd1 homodimers in the head-to-tail helical polymer are colored blue and green, respectively; the interacting molecule is colored black. The three residues, Arg400, Arg427, and Glu468, involved in the formation of electrostatic bonds are highlighted and shown as stick models. The double-helical mCcd1 polymer blocks the formation of electrostatic bonds between the three residues by inserting loop β1-β2 into the head-to-tail interface. (**f**) Residues involved in the formation of the zCcd1 single-helical filament. Electrostatic bonds are indicated by black dashed lines. Arg368, Arg395, and Glu436 (highlighted) form two electrostatic bonds in the head-to-tail interface. (**g**) Superposition of mCcd1 and zCcd1 homodimers.

A double-helical polymer of the mCcd1 DIX domain is assembled via zipper-like interactions between two head-to-tail helical dimers by the insertion of loop β1-β2 into Site 2 of head-to-tail interface in crystals. The mCcd1 DIX domain forms contacts with four adjacent DIX domains of another head-to-tail helical polymer through two types of interactions (Fig. 3a). The first and fourth contact sites present type I interaction between the hydrogen bond of Arg400 (loop β1-β2) and the carbonyl group of Phe442 (loop β3-β4) (Fig. 3b). The second and third sites present type II interaction between sheet β1-β5-β3-β4 and loop β1-β2 (Fig. 3c), where a salt bridge is formed by Asp399 (loop β1-β2) and Lys446 (strand β4). Furthermore, the carbonyl group of Asp399 forms a hydrogen bond with Trp466 (strand β5). Three residues, Phe397, Trp466, and Glu468, make hydrophobic contacts with side chains of Asp399, Arg400, and Leu402. The average total accessible surface area buried by type II interface is 286 Å^2^. The structural features of the double helical polymer formed by mCcd1 DIX suggest a possibility that the ability of mCcd1 for homopolymerization is weakened by the molecular interactions observed only in the double helical polymer.

To elucidate the molecular mechanism underlying the weakened ability of mCcd1 for homopolymerization, we performed superposition of head-to-tail homomeric dimers (Fig. 3d). Comparison of mCcd1 and zCcd1 DIX domains revealed rotational differences of 39.0° caused by type II interactions in double-helical polymers, which involved the insertion of loop β1-β2 into Site 2 interface of head-to-tail contacts (Fig. 3e). On the other hand, Site 2 interface in zCcd1 was stabilized by two electrostatic interactions of Glu436 with Arg368 and Arg395 in the single-helical filament, despite residue conservation in loop β1-β2 of mCcd1 (Fig. 3f). These findings suggest that mCcd1 DIX may destabilize the head-to-tail helical polymer by inhibiting the head-to-tail contact via type II interactions (Fig. 3g).

### Identification of residues determining low ability of Ccd1 DIX for homopolymerization

**Figure 4:**
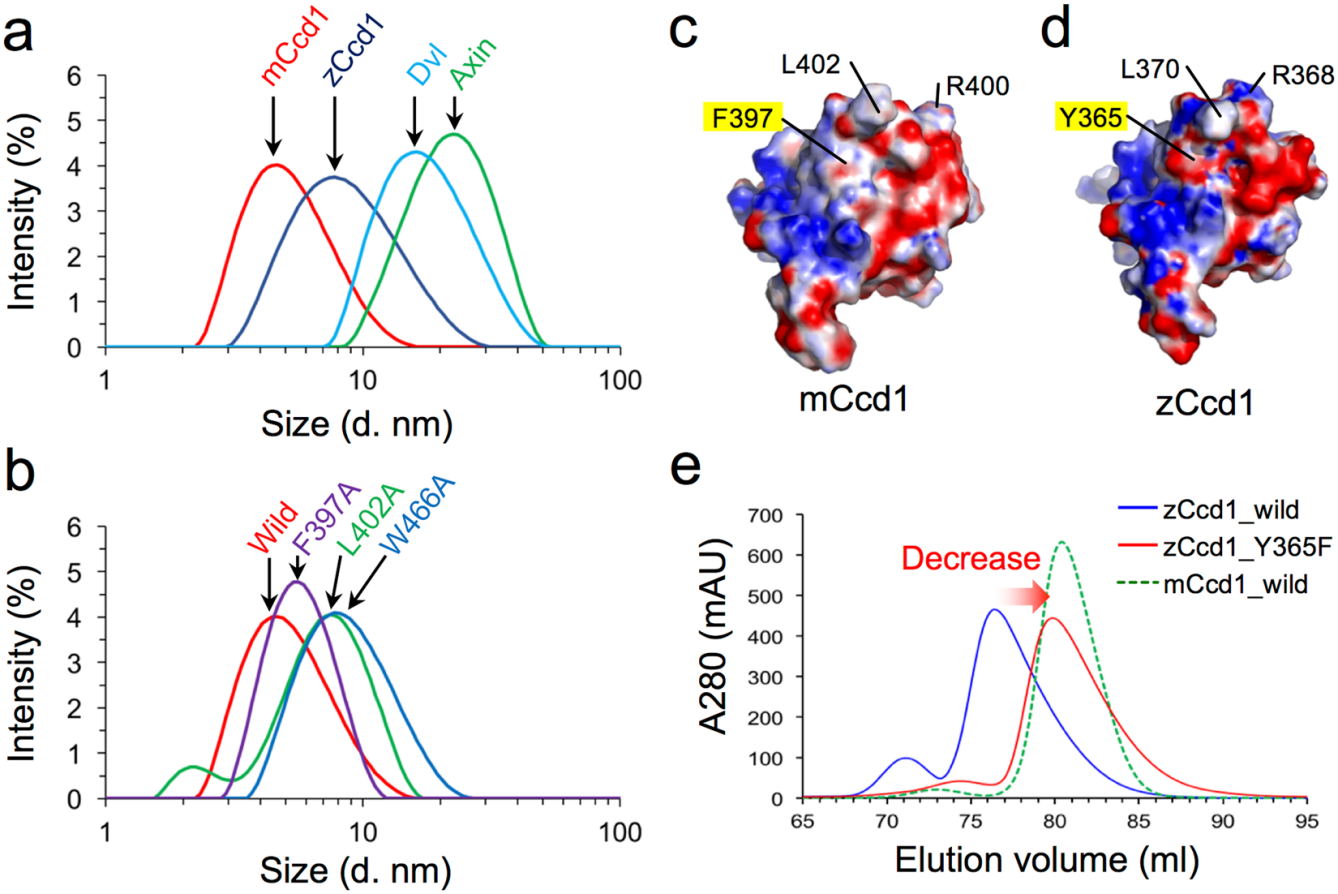
Residues responsible for mCcd1 auto-inhibition of homopolymerization. (**a**) Estimation of the apparent molecular size of mCcd1, zCcd1, Dvl1, and Axin DIX domains at the concentration of 100 μM by dynamic light-scattering. (**b**) Comparison of mCcd1 mutants carrying indicated substitutions of amino acids located at the contact interface for the double-helical polymerization. (**c**,**d**) Surface electrostatic potential of molecular interfaces in double-helical polymerization of mCcd1 DIX (**c**) and single-helical polymerization of zCcd1 DIX (**d**) viewed from the same direction. Positive (blue) and negative (red) potentials are indicated. Two conserved residues, Leu and Arg, in loop β1-β2 and the side chains of Phe397 (mCcd1) and Tyr365 of (zCcd1) are indicated. (**e**) The effect of Y365F substitution on zCcd1 homopolymerization state. Gel filtration profiles of DIX domains of zCcd1 (wild-type and Y365F mutant) and mCcd1 (wild-type) are shown.

To compare the ability for homopolymerization in DIX domains of mCcd1, zCcd1, Dvl1, and Axin, we analyzed their physicochemical properties by dynamic light scattering (DLS). DIX domain polymers were observed as single peaks, although size distribution was not monodisperse (Fig. 4a): average diameters of polymers formed by mCcd1, zCcd1, Axin, and Dvl1 DIX domains at the concentration of 100 μM were 4.67 ± 2.1, 7.76 ± 4.4, 22.45 ± 8.0, and 16.24 ± 7.4 nm, respectively. The size of the mCcd1 DIX homopolymer was similar to that reported previously and corresponded to the width of a head-to-tail dimer in the double-helical polymer^17, 37^, whereas the DIX domain of zCcd1 formed a homopolymer containing a single helical turn. The ability of homopolymerization in Ccd1 DIX was weaker than those of DIX domains in Dvl1 and Axin.

To test whether the ability for homopolymerization in mCcd1 DIX was weakened by the insertion of loop β1-β2 into the head-to-tail interface, we generated five mutant proteins in which amino acids in the contact interface of the double-helical polymer were replaced with Ala. Interestingly, two mutations, L402A and W466A, in mCcd1 DIX increased the molecular diameter of the homopolymer compared with that of the wild type (Fig. 4b). The W466A substitution in strand β5 resulted in the highest increase in polymer molecular size: up to 7.76 ± 3.6 nm in diameter. The substitution of the hydrophobic Leu402 residue (loop β1-β2) also affected polymer formation as evidenced by the increase of its diameter to 7.41 ± 2.6 nm. It should be mentioned that the molecular sizes of the L402A and W466A mutants were comparable to that of zCcd1 DIX (Fig. 4a). In contrast, mutants carrying F397A, D399A, and R400A substitutions did not demonstrate significant size changes (Fig. 4b and Fig. S3a). These results indicate that Leu402 and Trp466 play an important role in mediating the ability for homopolymerization in mCcd1 DIX.

Although the low ability of mCcd1 DIX for homopolymerization is mediated by the insertion of loop β1-β2 into the head-to-tail interface, the key Leu402 and Trp466 residues are also conserved in zCcd1 (Fig. 1a), suggesting that loop β1-β2 of zCcd1 DIX could not make contact with the head-to-tail interface of the dimer. Phe397 of mCcd1, which is close to the contact surface with loop β1-β2, is replaced with Tyr365 in zCcd1 (Fig. 1a and Fig. S3b), thereby changing the molecular surface from hydrophobic in mCcd1 to negatively charged in zCcd1 (Fig. 4c,d). The substitution of Tyr365 with Phe in zCcd1 DIX decreased the apparent molecular weight of the mutant compared to the wild type molecule, making it comparable with that of mCcd1 DIX (Fig. 4e). This result suggests that the molecular surface of the Ccd1 DIX domain plays a key role in defining specific polymerization state.

### Heteromeric interactions of mCcd1 DIX in auto-inhibitory mode

**Figure 5:**
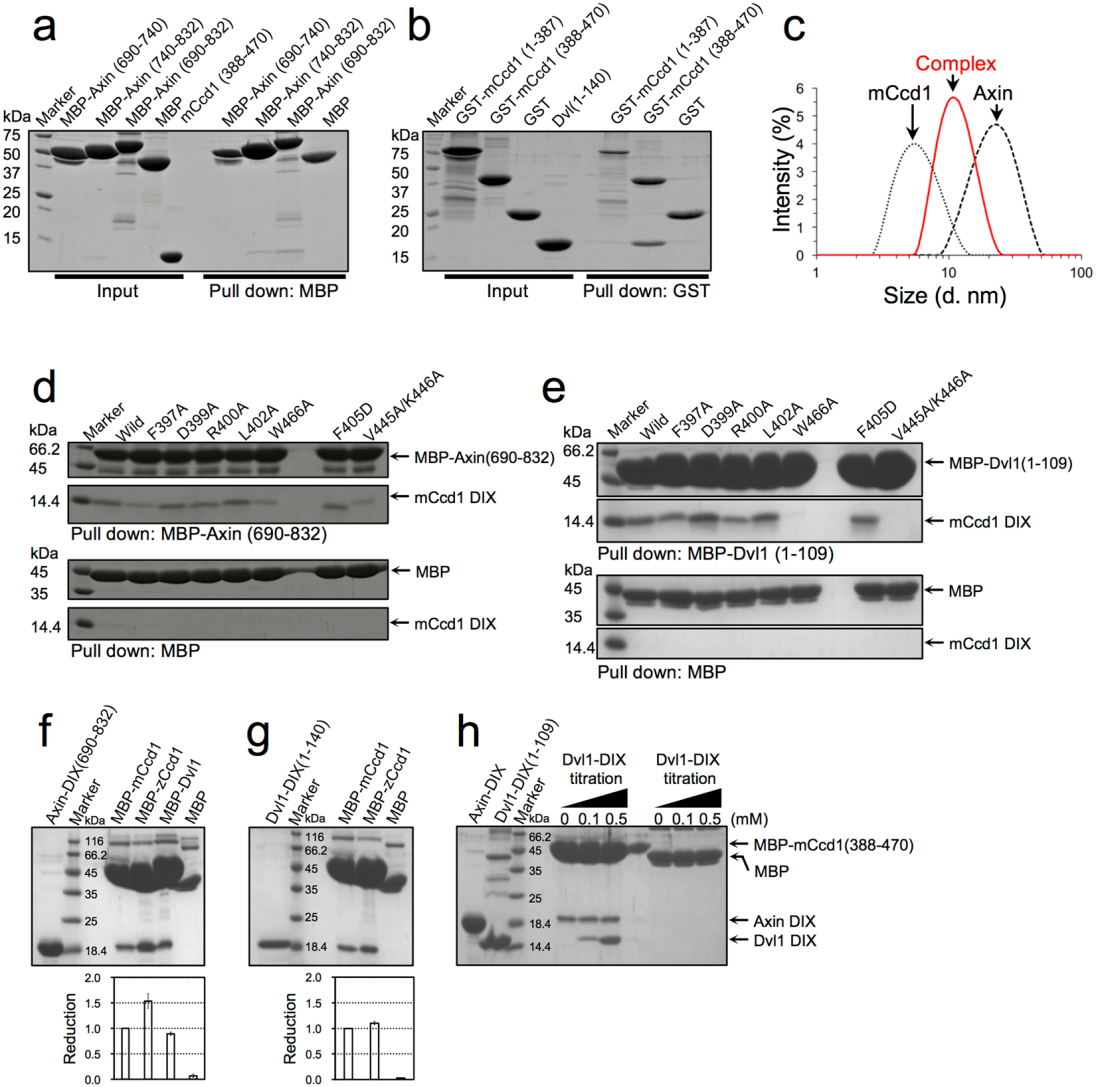
Heteromeric DIX-DIX interactions between mCcd1, Axin, and Dvl. (**a**) Pull-down assay of mCcd1 with MBP-fused Axin fragments. (**b**) Pull-down assay of Dvl1 with GST-fused mCcd1 fragments. (**c**) Apparent molecular size of the heteromeric complex (red line) formed between DIX domains of mCcd1 (dotted line) and Axin (dashed line) at the concentration of 100 μM. (**d**,**e**) Pull-down assay of mCcd1 (wild type and mutants) with MBP-fused Axin (**d**) and Dvl1 (**e**) (The full-length gel images are available in supplementary information Fig. S6). (**f**) Pull-down assay of the MBP-fused mCcd1, zCcd1, and Dvl1 (0.8 mM) with Axin. (**g**) Pull-down assay of the MBP-fused mCcd1 and zCcd1 (0.8 mM) with Dvl1. (**h**) Effect of Dvl1 on Axin binding to Ccd1. The pull-down assay of the MBP-fused mCcd1 DIX domain with Axin DIX domain was performed without or with Dvl1 DIX at the indicated concentrations. Axin-mCcd1 binding was not influenced by Dvl1.

The formation of the Ccd1-Axin complex depends on heteromeric DIX-DIX interactions which are critical for the Ccd1-dependent activation of the canonical Wnt pathway^15^. Therefore, we analyzed the formation of heteropolymers between DIX domains of mCcd1 and Axin by the pull-down assay using purified MBP-tagged proteins. The binding to mCcd1 DIX was detected for Axin constructs containing residues 690–832 or 740–832, but not for that containing residues 690–740 (Fig. 5a). Similarly, the pull-down assay confirmed the interaction between DIX domains of Dvl1 and mCcd1 (Fig. 5b), which is consistent with previous reports^17, 33^. DLS detected a heteromer formed by DIX domains of mCcd1 and Axin (mixed in solution at the 1:1 molar ratio) as a single peak corresponding to a molecular size of 10.72 ± 4.2 nm, which is smaller than that of a homopolymer formed by Axin DIX (Fig. 5c). Similarly, the molecular weight of Dvl1-mCcd1 and Dvl1-Axin heteromers corresponded to the combined weight of respective polymers (Fig. S4). Furthermore, the DIX-binding between mCcd1 and Axin reduced homopolymer formation of Axin DIX domain (Fig. S5). These results indicate that DIX domains of Ccd1, Axin, and Dvl1 form heteromeric structures through direct binding, confirming their interactions in the β-catenin destruction complex.

Next, we examined whether the contact interface in double-helical homopolymer of the mCcd1 DIX domain was involved in the formation of heteropolymers with DIX domains of Axin and Dvl1 by the pull-down assay using mutant proteins (Fig. 5d,e). Compared with the wild-type protein, mutants F397A and W466A had reduced affinity to Axin DIX, while substitutions of residues located at loop β1-β2 (D399A, R400A, and L402A) did not change the binding affinity. Furthermore, we generated other mutants carrying mutations in the homomeric head-to-tail interface of mCcd1 DIX^32^ (Fig. 1a). The V445A/K446A double mutation significantly reduced the binding affinity of mCcd1 to Axin, while the F405D mutation caused no changes compared to the wild type. We also examined the effect of amino acid substitutions in mCcd1 DIX on its binding to Dvl1 DIX (Fig. 5e). W466A and V445A/K446A mutations completely abolished mCcd1 DIX binding to the Dvl1 DIX domain. These results indicate that the hydrophobic surface formed by the two aromatic residues (Phe397 and Trp466) on the β-sheet and the tail region of mCcd1 DIX contributed to heteropolymerization with DIX domains of Axin and Dvl, whereas loop β1-β2 responsible for the reduction of mCcd1 DIX ability to homopolymerization was not involved.

To determine the functional significance of the inhibition of mCcd1 homopolymerization by loop β1-β2, we compared the binding affinity of mCcd1 and zCcd1 to Axin and Dvl1, respectively (Fig. 5f,g). The DIX domain of zCcd1 displayed 1.53-fold stronger binding to Axin at high concentration (0.8 mM) compared to that of mCcd1, whereas there was no difference in their affinity to Dvl1. These results indicate that the inhibition of mCcd1 homopolymerization decreased its heteropolymerization with Axin, but not with Dvl1. Furthermore, we investigated the effect of Dvl1 on heterotypic binding between DIX domains of mCcd1 and Axin (Fig. 5h). The presence of Dvl1 DIX did not enhance the association between DIX domains of Axin and mCcd1, although heteromeric DIX-mediated binding between Dvl1 and mCcd1 increased in a concentration-dependence manner. The results suggest that the DIX domain of Dvl1 did not cooperatively promote the binding between DIX domains of Axin and mCcd1.

### Mutations blocking mCcd1 DIX auto-inhibition increase Wnt signaling *in vivo*

**Figure 6:**
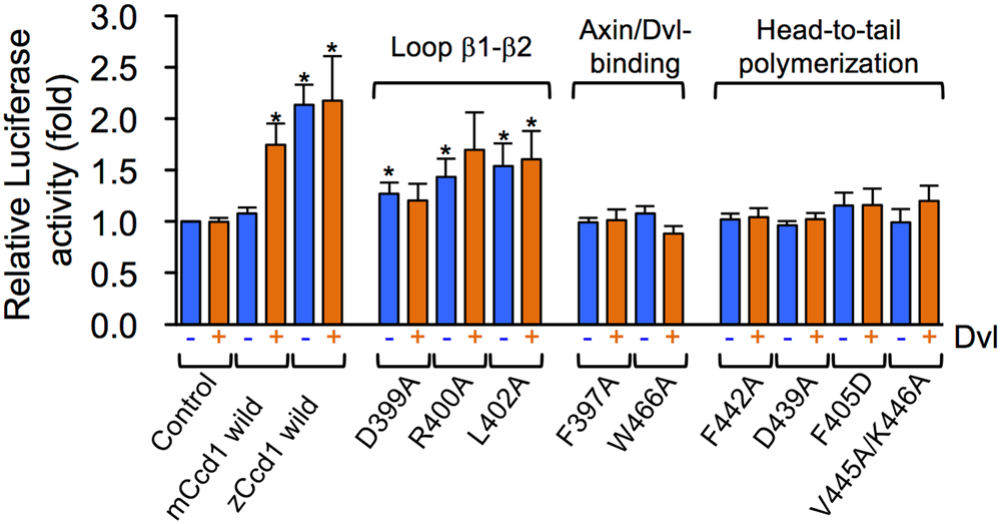
Effect of homopolymerization on mCcd1 signaling activity. TCF-dependent transcriptional activity in HeLa cells transfected with mCcd1 (wild type and mutants) and zCcd1 was analyzed by a luciferase reporter assay. The data are shown as the means ± standard error based on five independent experiments. Asterisks indicate statistical significance compared with negative control in the absence of Dvl (p < 0.05 by Student’s *t*-test).

Using a panel of mCcd1 mutants, we examined whether the identified contact sites involved in the inhibition of mCcd1 DIX homopolymerization through loop β1-β2 were relevant to the activation of Wnt signaling. The involvement of Ccd1 in the canonical Wnt pathway was assessed by transcriptional activation in the Wnt-specific TOPflash luciferase reporter assay^38^ (Fig. 6). Although the transfection with FLAG-tagged BαL isoform of mCcd1 alone induced little TCF-dependent transcription, co-expression with low levels of Dvl1, which did not significantly induce the reporter activation by itself, increased transcriptional activity by 1.7-fold via a synergistic effect^36^. In contrast, zCcd1 alone caused significant (2.1-fold) transcriptional activation of the reporter, but synergy with Dvl was not observed^15^. The mCcd1 mutants carrying D399A, R400A, and L402A substitutions in loop β1-β2 caused 1.3-, 1.4- and 1.5-fold transcriptional activation, respectively, compared to the wild type. However, the transcriptional activity of these mutants did not demonstrate synergy with Dvl1. Thus, replacement of amino acids responsible for auto-inhibition of homopolymerization in mCcd1 DIX converted it to a constitutively active form demonstrating molecular mode similar to that of zCcd1, which could alone activate TCF-dependent transcription.

Next, we examined the functional significance of mCcd1 heteromerization with Axin and Dvl1 through DIX domains. Substitutions of Phe397 and Trp466 located at the β1-β5 sheet involved in the heteromeric interaction with Axin and Dvl1 and mutations of the residues required for head-to-tail homomerization (F442A, D439A, F405D, and V445A/K446A) decreased the transcriptional activity of mCcd1 both alone and in synergy with Dvl (Fig. 6). These results suggest that the molecular surface created by hydrophobic residues of the β1-β5 sheet plays an important role in the transcriptional activation of the Wnt signaling pathway via heteropolymerization with DIX domains of Axin and Dvl.

## Discussion

DIX domains of Dvl, Ccd1, and Axin are essential for the formation of the β-catenin destruction complex, where they regulate β-catenin stability and transcriptional activation^13, 15, 39^. The overexpression of zCcd1 and Dvl is known to activate Wnt signaling^15, 22^, while that of mammalian Ccd1 hardly induces β-catenin-dependent transcription^16, 17, 36^. These findings support an assumption that mCcd1 signaling activity is restricted by a specific structural state distinct from that of zCcd1 and Dvl. Although the co-expression of Ccd1 with Dvl exerts a synergistic effect on transcriptional activity, the regulatory mechanism of Ccd1 function remains unknown^17, 36^. However, the observation that dominant-negative Ccd1 inhibits transcriptional activation induced by overexpressed full-length Ccd1 and Dvl *in vivo* suggests the importance of DIX-dependent homomeric and heteromeric polymerization in Wnt signaling^15, 36^.

Our physicochemical analysis showed that the mCcd1 DIX domain could not form homopolymers of high molecular weight, indicating weak capacity for self-polymerization compared with zCcd1 DIX domain (Figs 1c,d and 4a). Furthermore, structural and mutational analyses revealed that mCcd1 DIX domain auto-inhibits the propensity to form homopolymers by the insertion of loop β1-β2 into head-to-tail interface. Although the zCcd1 DIX domain displays high sequence similarity to mCcd1 DIX (78% identity), hydrodynamic properties of the DIX domain in zCcd1 are similar to those of Dvl and Axin rather than to that of mCcd1. Interestingly, we found that the functional difference between DIX domains of mCcd1 and zCcd1 is underlain by the replacement of an aromatic residue located at the molecular surface which is in contact with loop β1-β2 during inhibition of the head-to-tail interaction (Fig. 4c–e). The residues involved in the auto-inhibition of mCcd1 DIX are not conserved in DIX domains of Dvl1 and Axin (Fig. 1a). These findings suggest that mCcd1 could suppress its signaling activity in the Wnt pathway by auto-inhibiting homopolymerization.

The analysis of mCcd1 binding to Axin and Dvl1 indicates that the residues in the interface of both head-to-tail and auto-inhibitory interactions contribute to the assembly of heteropolymers (Fig. 5d,e). This observation is consistent with previous findings that head-to-tail interface is involved in the heteromerization of Dvl with Axin and Ccd1 through direct interaction^17, 33^. Importantly, the W466A mutation in mCcd1 DIX resulted in the reduction of transcriptional activity in the reporter assay due to decreased binding affinity to the Axin DIX domain; at the same time, the propensity toward head-to-tail homopolymerization increased. Our structural analysis indicates that in mCcd1, the side chain of Trp466 would stabilize the conformation of the Lys446 side chain involved in the head-to-tail interaction and homomerization (Fig. 2d). In addition, a previous study showed that the mutation of Lys444 in hCcd1, which corresponds to Lys446 in mCcd1, resulted in a significant reduction of DIX binding to Dvl1^17^. The replacement of Trp466 with Ala may affect DIX conformational state required for heteromerization, although we cannot exclude the possibility that this residue is engaged in the contact interface of the heteropolymeric complex.

The difference between DIX-dependent homopolymerization in mCcd1 and zCcd1 uncovers the molecular regulatory mechanism of mammalian Ccd1 activation in Wnt signaling, which may be based on promoting single-helical polymerization by removing the auto-inhibitory effect. Although mCcd1 activity in the Wnt pathway was enhanced by co-expression with Dvl^17, 36^, mCcd1-Dvl heteropolymerization through DIX domains did not promote mCcd1 binding to Axin, suggesting that the synergy between Ccd1 and Dvl may be mediated by regulatory mechanisms distinct from heteromerization. A recent study indicated that the Ccd1 AαL isoform stimulated proliferation of neuronal progenitor cells by upregulating β-catenin-dependent transcription through direct binding of Disrupted in Schizophrenia-1 (DISC1), a psychiatric illness risk gene playing an important role in brain development^40, 41^, to the region between the calponin homology (CH) domain and coiled-coil (CC) motif^16^. CC motifs function as allosteric regulatory modules via rearrangement of axial positions of the two helices, which may affect ATP hydrolysis and protein-protein interactions^42–45^. It is possible that Ccd1 binding to DISC1 in the area adjacent to the CC motif may affect polymeric state of the DIX domain via long-distance allosteric change, which can be one of the mechanisms for Ccd1 activation.

In addition, Ccd1 activity may be regulated by protein kinases. Thus, the phosphorylation of Ser250 at the N-terminal region of the Ccd1 AαL isoform by cyclin-dependent kinase 5 (Cdk5) facilitates its interaction with DISC1-binding partner NdelI, promoting neuronal migration^16^. However, Cdk5-mediated Ccd1 phosphorylation did not affect Wnt signaling. Furthermore, Ser593 located in the DIX domain of the AαL isoform was identified as another phosphorylation site targeted by Microtubule Affinity-Regulating kinases MARK1/4^46^. MARK1/4-phosphorylated Ccd1 bound 14–3–3 scaffolding proteins and localized at focal adhesions, negatively regulating cell migration and invasion, while S593A mutation promoted cell invasion and wound healing. Although the effect of MARK1/4 phosphorylation on Ccd1 signaling activity in the Wnt pathway has not been investigated, it is possible that the binding of 14–3–3 proteins to the phosphorylated Ccd1 in the vicinity of the DIX domain may produce conformational changes and promote the single-helical polymerization in Ccd1. Further studies are needed to determine the effect of phosphorylation on the regulation of Ccd1-dependent activation of the Wnt pathway.

Finally, our study suggests a possibility that zCcd1 underwent evolution in a unique way. The genome sequence analysis indicated that an average of 2.28 genes in zebrafish are associated with each human orthologous gene, because this organism underwent an additional round of whole-genome duplication called the teleost-specific genome duplication (TSD)^47, 48^. The genome search using the zCcd1 sequence revealed that zebrafish possesses another homologous gene, zCcd2 (Gene ID: 794716)^49^. The zCcd2 gene would generate two isoforms, zCcd2A and zCcd2B, by usage of multiple transcription start site, displaying 61.5% and 61.0% of the amino acid sequence identity with Abeta2L and BalphaL of mCcd1 isoforms. Interestingly, the zCcd2 DIX domain shows the high similarity to that of mCcd1 (86.7%) rather than zCcd1 (84.3%) and has a Phe residue at the position corresponding to Phe397 of mCcd1. Therefore, zCcd2 alone would not activate β-catenin-dependent transcription due to low affinity for the DIX-homopolymerization and thus play a similar role to that of mCcd1 in the Wnt signaling pathway. These findings suggest that zCcd1 is a unique gene evolved after TSD in zebrafish for efficient activation of the Wnt signaling pathway in embryonic development. On the other hand, the higher organisms may be complement by the splicing isoforms of Ccd1 generated from a unique gene, although the activation mechanism of mCcd1 remains as unknown. Further work is required for understanding the physiological role of two homologous Ccd1 proteins in zebrafish and the regulatory mechanism of individual Ccd1 subtypes of mammalian species.

## Experimental Procedures

### Sample preparation

DIX domains of mCcd1, Axin, and Dvl1 were expressed as fusion constructs with glutathione S-transferase (GST) or maltose-binding protein (MBP) in *Escherichia coli*. DIX domains of mCcd1 was purified and crystallized as described earlier^37, 50^. Mutants were constructed using the QuickChange Site-Directed Mutagenesis kit (Agilent Technologies) and confirmed by DNA sequencing.

Nucleotide fragments encoding the DIX domain of zCcd1 were amplified by PCR using primers designed based on cDNA (Gene ID 360137) and the amplified fragment was subcloned into the pOPINM plasmid (Addgene) using the in-Fusion HD cloning technology kit (Clontech Laboratories, Inc.)^51^. Cells expressing the MBP-fused zCcd1 DIX domain were suspended in 50 mM Na HEPES buffer (pH 7.5) containing 150 mM NaCl, 1 mM dithiothreitol (DTT), and 1% TritonX-100 and disrupted by ultrasonication at 277 K. The supernatant was loaded onto an amylose resin column (New England Biolabs) and the MBP-fused zCcd1 DIX was eluted with 50 mM HEPES-Na (pH 7.5) containing 150 mM NaCl, 1 mM DTT, and 20 mM maltose. To cleave the MBP tag, the purified MBP-fused zCcd1 DIX domain was incubated with an excess molar ratio of the GST-fused HRV3C protease at 284 K overnight and applied to a HiTrap Q high performance column (GE Healthcare) equilibrated with a loading buffer consisting of 20 mM HEPES-Na (pH 7.5), 150 mM NaCl, and 1 mM DTT. The column was washed with the loading buffer and the protein was eluted using a linear NaCl gradient (0.15–0.5 M).

### Crystallization and data collection

The mCcd1 DIX domain was crystallized as described earlier^37^. To achieve crystallization, the purified zCcd1 DIX was concentrated to 2.4 mg ml^–1^ in a solution containing 10 mM HEPES-Na (pH 7.5), 150 mM NaCl, and 1 mM DTT using an Amicon 20 concentrator (5 K MWCO PS, Vivascience). Crystallization was performed by the sitting-drop vapor-diffusion method at 283 K. The crystals of zCcd1 DIX were obtained in solution containing 1.2 mg ml^–1^ protein, 55 mM HEPES-Na (pH 7.5), 7% poly(acrylic acid sodium salt) 5100, 10 mM MgCl_2_, 5 mM TCEP-HCl, 75 mM NaCl, and 0.5 mM DTT equilibrated against 14% poly(acrylic acid sodium salt) 5100, 100 mM HEPES-Na (pH 7.5), 20 mM MgCl_2_, and 10 mM TCEP-HCl at 283 K. The crystals were soaked in cryoprotective solution containing 14% poly(acrylic acid sodium salt) 5100, 100 mM HEPES-Na (pH 7.5), 20 mM MgCl_2_, 10 mM TCEP-HCl, and 15% glycerol and flash-cooled in liquid nitrogen. X-ray diffraction was performed using the BL-1A beamline at the Photon Factory, Tsukuba, Japan with a DECTRIS PILATUS 2M-F detector. All data were processed and scaled using the HKL2000 program^52^.

### Structural determination

Based on the MAD dataset of SeMet crystals of the mCcd1 DIX domain, seven selenium positions were determined using SHELX-D^53^. Phasing and density modifications were performed using SHARP^54^ and SOLOMON^55^, respectively, and models were built by the COOT graphic program^56^. The structure was refined at 3.00 Å resolution by simulated annealing and TLS refinement methods using the PHENIX program^57^. The refined model was used to determine the structure of the zCcd1 DIX domain by the molecular replacement method using the PHASER program^58^; the obtained structure was refined at 1.96 Å using PHENIX. Stereochemical quality of the models was verified using the MolProbity program^59^. The refinement statistics are summarized in Table 1. Atomic coordinates and structural factors for the mCcd1 and zCcd1 DIX domains have been deposited in the Protein Data Bank under ID code 5Y3B and 5Y3C﻿, respectively.

### Dynamic light-scattering measurements

DLS measurements were carried out using Zetasizer Nano (Malvern Instruments Ltd). Protein samples were centrifuged at 20,000 × *g* for 10 min to remove debris, and measurements were performed at 293 K using a 50 μL quartz cuvette filled with 50 μL protein solution. Size distributions were calculated based on intensity data acquired at regular time intervals.

### Pull-down assay

Each sample was incubated at 277 K for 1 h in pull-down buffer (10 mM HEPES-Na, pH 7.5, 150 mM NaCl, 1 mM DTT), and complexes were precipitated using amylose resin equilibrated with pull-down buffer. The bound complexes were washed three times with 1.0 ml of pull-down buffer and eluted with 30 μL of pull-down buffer supplemented with 20 mM maltose or glutathione. The amount of eluted MBP- or GST-fusion proteins and bound DIX domains was determined by SDS-PAGE.

### Luciferase reporter assay

Transcriptional activity of Ccd was analyzed by the Wnt-specific TOPflash luciferase reporter assay. HeLa cells grown in 24-well plates with Minimal Essential Medium (MEM) were transfected with 200 ng of the firefly luciferase reporter plasmid, 10 ng of the *Renilla* luciferase plasmid (internal control), and 200 ng of Ccd1 in the absence or presence of 10 ng *Xenopus* Dvl using FuGENE HD (Roche) according to the manufacturer’s instructions. The total DNA amount in each well was adjusted to 600 ng with β-galactosidase cDNA. Luciferase activity was measured by the dual-luciferase reporter assay system (Promega), and the activity of firefly luciferase was normalized to that of *Renilla* luciferase.

## Electronic supplementary material

Supplemental information
