# Supplementary material for: Structural basis for Ccd1 auto-inhibition in the Wnt pathway through homomerization of the DIX domain
Source: Sci Rep. 2017 Aug 10;7:7739. doi: 10.1038/s41598-017-08019-5 (PMC5552852; doi:10.1038/s41598-017-08019-5)
Supplement: Supplementary file 1 — Supplemental information [file 41598_2017_8019_MOESM1_ESM.pdf]

Supplemental information

Structural basis for Ccd1 auto-inhibition in the Wnt pathway through homomerization of the DIX domain

Shin-ichi Terawaki[1,2*], Shohei Fujita[1], Takuya Katsutani[3], Kensuke Shiomi[4], Kazuko Keino-Masu[4], Masayuki Masu[4], Kaori Wakamatsu[1], Naoki Shibata[2,3] & Yoshiki Higuchi[2,3*]

[1] Graduate School of Science and Technology, Gunma University, 1-5-1 Tenjin-cho, Kiryu, Gunma 376-8515, Japan

[2] RIKEN SPring-8 Center, 1-1-1 Koto, Mikazuki-cho, Sayo-gun, Hyogo 679-5248, Japan

[3] Department of Life Science and Department of Picobiology, Graduate School of Life Science, University of Hyogo, 3-2-1 Koto, Kamigori-cho, Ako-gun, Hyogo 678-1297, Japan

[4] Department of Molecular Neurobiology, Faculty of Medicine, University of Tsukuba, 1-1-1 Tennodai, Tsukuba, Ibaraki 305-8575, Japan

***Corresponding authors:**

Yoshiki Higuchi

Department of Life Science, University of Hyogo, 3-2-1 Koto, Kamigori-cho, Ako-gun, Hyogo 678-1297, Japan

Tel: +81-791-58-0179; Fax: +81-791-58-0177; E-mail: hig@sci.u-hyogo.ac.jp

Shin-ichi Terawaki

Graduate School of Science and Technology, Gunma University, 1-5-1 Tenjin-cho, Kiryu, Gunma 376-8515, Japan

Tel.: +81-277-30-1449; Fax: +81-277-30-1439; E-mail: terawaki@gunma-u.ac.jp

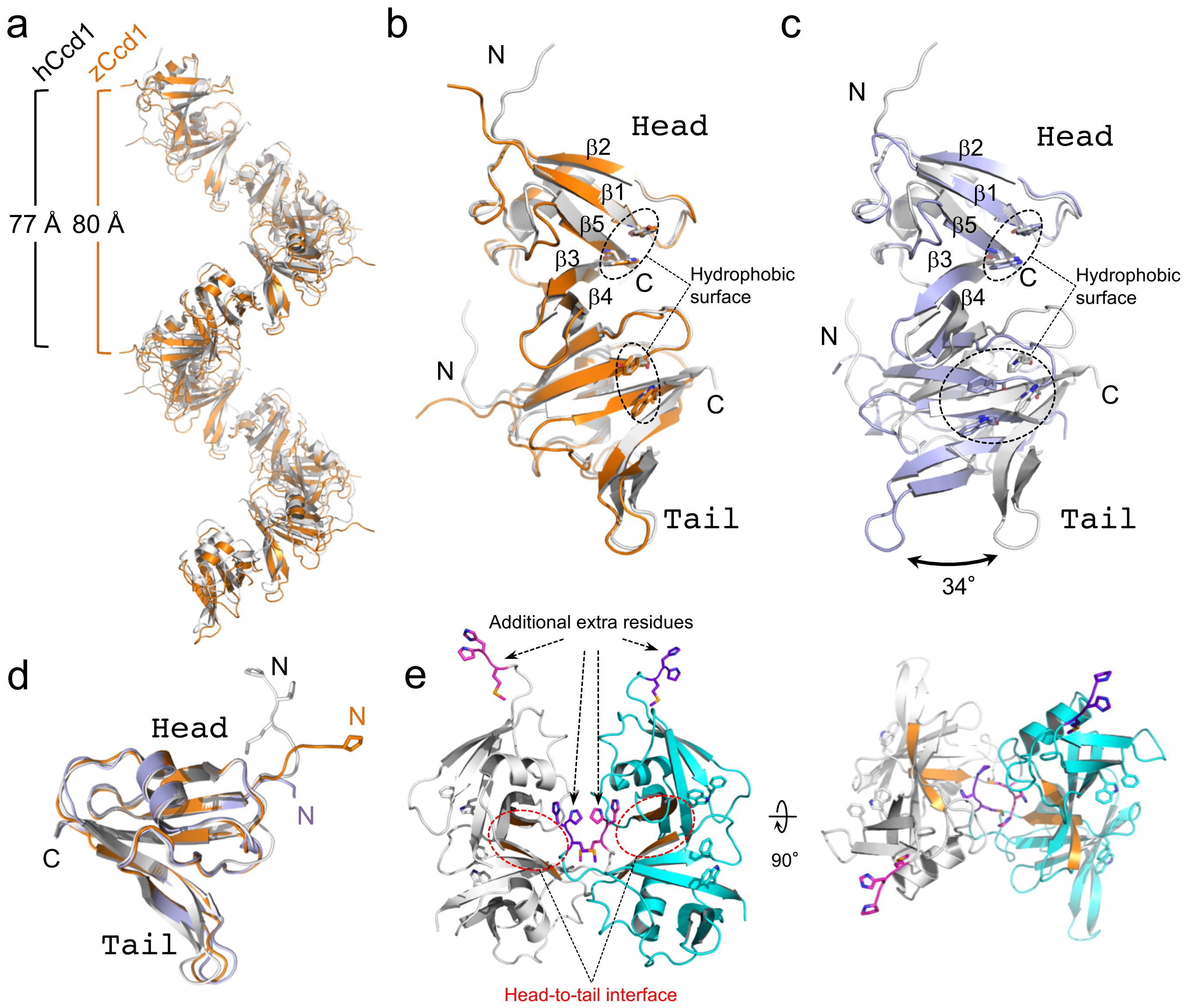

Figure S1. Structural comparison of mouse and zebrafish Ccd1 DIX domains with the human Ccd1 DIX domain. (a) Superposition of zCcd1 (orange) and hCcd1 (white) DIX domains. Top molecules of each helical polymer are used for superposition; helical pitches are indicated. **(b)** Superposition of zCcd1 dimer (orange) and hCcd1 dimer (white). Top molecules of each head-to-tail dimer are used for superposition; aromatic residues in the hydrophobic interface of double-helical polymerization are circled. **(c)** Superposition of mCcd1 dimer (light blue) and hCcd1 dimer (white). The angular difference between the dimers is indicated by a double-headed arrow. **(d)** Superposition of mCcd1 (light blue), zCcd1 (orange), and hCcd1 (white) DIX domains with distinct conformations of the N-terminus. **(e)** Crystal structure of the hCcd1 DIX tetramer. Additional residues in the N-terminus are shown as stick models colored magenta and purple; parallel β-bridges in the head-to-tail interface are indicated by red circles.

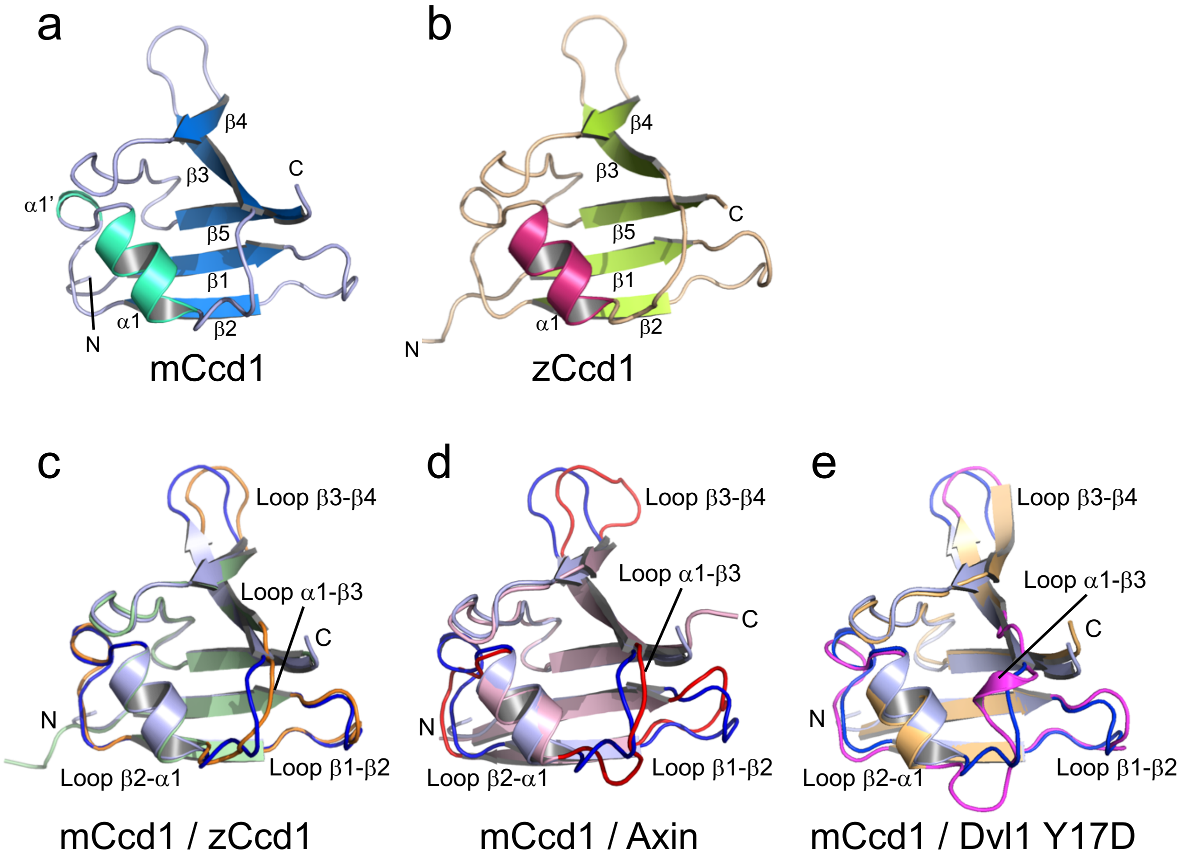

Figure S2. The structure of the DIX domain monomers. (a, b) Structures of DIX domain monomers: (a) mCcd1 and (b) zCcd1. **(c)** Superposition of mCcd1 (light blue) and zCcd1 (green) DIX monomers. The four-loop regions of the mCcd1 and zCcd1 DIX domains are colored blue and orange, respectively. **(d)** Superposition of mCcd1 (light blue) and Axin (purple) DIX monomers. The four loop regions of the mCcd1 and Axin DIX domains are colored blue and red, respectively. **(e)** Superposition of DIX monomers of mCcd1 (light blue) and Dvl1 Y17D mutant (yellow). The four loop regions of the mCcd1 and Dvl1 mutant are colored blue and magenta, respectively.

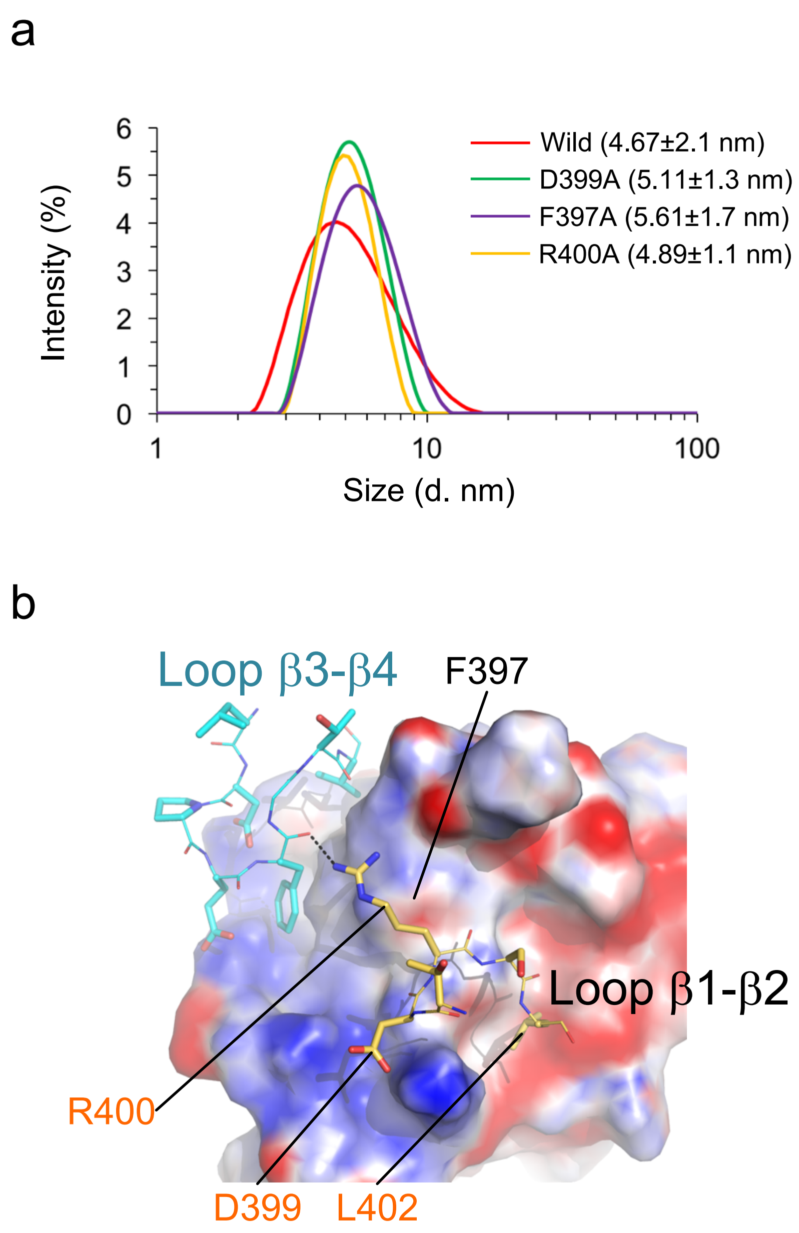

Figure S3. Identification of residues involved in mCcd1 double-helical polymerization. (a) Dynamic light scattering analysis of the wild-type and mutant (D399A, F397A, and R400A) mCcd1 DIX domains (100 μM). **(b)** Magnified view of the molecular surface in the interface of double-helical polymerization. Loops β1–β2 and β3–β4 are shown as stick models colored yellow and cyan, respectively. Molecular surface is colored red and blue for positive and negative, respectively, electrostatic potential; Phe397 position is indicated.

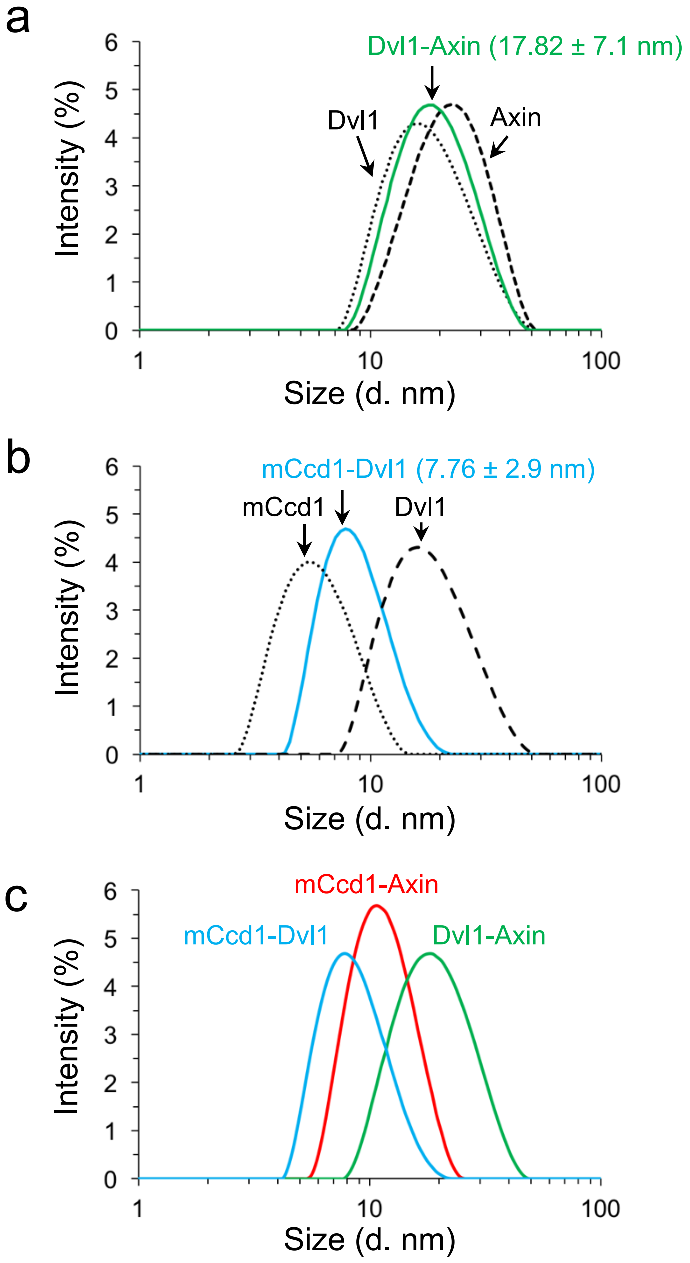

Figure S4. DLS analysis of heteropolymers formed by DIX domains of mCcd1, Dvl1, and Axin. (a) Apparent molecular size of the Dvl1-Axin heteropolymer (monomer concentration, 100 μM). **(b)** Apparent molecular size of the mCcd1-Dvl1 heteropolymer (monomer concentration, 100 μM). **(c)** Apparent molecular size of heteropolymers between mCcd1 and Axin, mCcd1 and Dvl1, and Dvl1 and Axin.

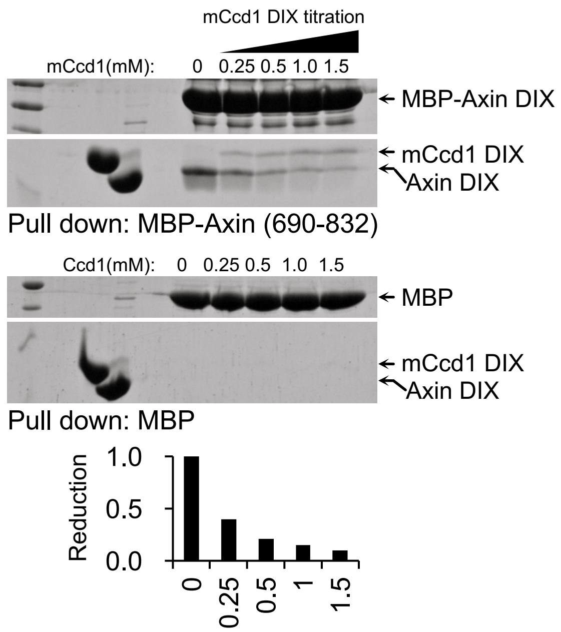

Figure S5. Effect of mCcd1 binding on homopolymerization of the Axin DIX domain. Homopolymerization between DIX domains of MBP-fused and non-tagged Axin was performed in the presence of the mCcd1 DIX domain at the indicated concentrations and analyzed by the pull-down assay. Axin homopolymerization was decreased in an mCcd1 concentration-dependent manner.

a

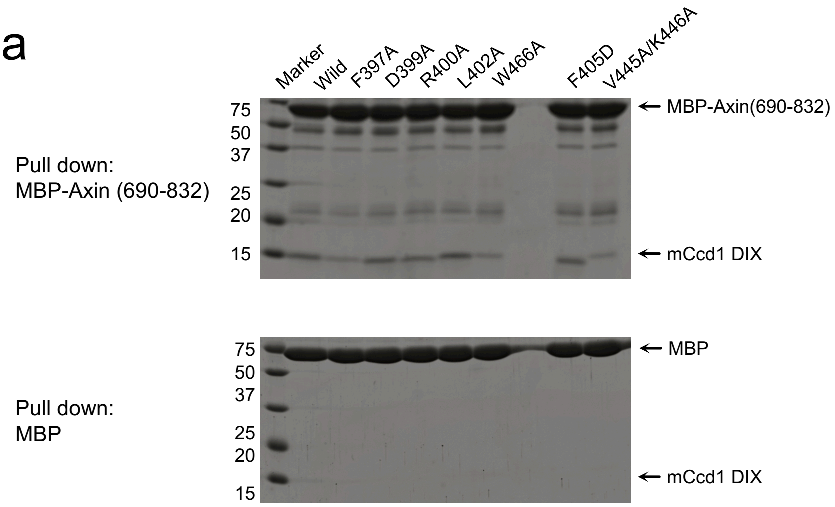

b

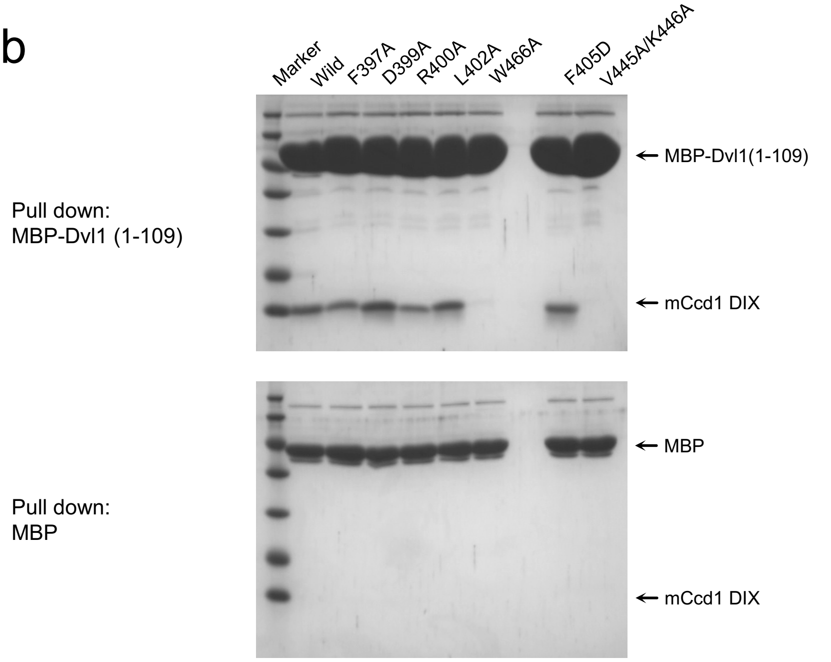

Figure S6. Full-length gel images of pull-down assay. Pull-down assay of mCcd1 (wild type and mutants) with MBP-fused Axin (a) and Dvl1 (b).
